# History of psychiatry in India

**DOI:** 10.4103/0019-5545.69195

**Published:** 2010-01

**Authors:** S. Haque Nizamie, Nishant Goyal

**Affiliations:** Central Institute of Psychiatry, Kanke, Ranchi - 834 006, Jharkhand, India

**Keywords:** History, India, psychiatry

## Abstract

History is a screen through which the past lightens the present and the present brightens the future. Psychiatry by virtue of its ability to deal with human thoughts and emotions and provide a pathway for healthy minds provides an important platform towards being a mentally sound human being and largely the society. This review takes a sneak peek into the foundations of modern psychiatry in India. The description is largely based on the time frame, which provides a better understanding of the factual information in each period starting from the Vedic era and culminating in the post independence period.

## INTRODUCTION

Mental Health by virtue of its ability to deal with human thoughts and emotions, and to provide a pathway for healthy minds is a vital resource for our development, and its absence represents a great burden to the economic, political, and social functioning of human beings, society and nation.[1] The scope of mental health is not only confined to the treatment of some seriously ill persons admitted to mental health centers, rather it is related to the whole range of health activities.[2] India has developed an endogenous, alternative body of knowledge which is more suited to Indian conditions.[3]

History is a screen through which the past lightens the present and the present brightens the future. The ancient Indian thought emphasized the theory of unity of body and soul and also explained how to deal with health and mental health problems in a psychosomatic way.[4] A concern with mental health has long been a part of Indian culture, which has evolved in a variety of ways, attempting to understand and negotiate psychological disorder.[5] This review takes a sneak peek into the foundations of modern psychiatry in India which has sailed through tides of time across the world.

## HISTORY OF WORLD PSYCHIATRY: A PRELUDE

The occurrence of mental illnesses has been identified and documented since ancient times. The earliest predecessor of mental hospitals on record was a Greek sanctuary at Epidauros. The fourth century AD witnessed the establishment of institutions solely for the mentally ill in Byzantium and Jerusalem.[6] Thereafter, Christian and Muslim religious orders established places of refuge for the mentally ill and patients were treated by a variety of procedures with a religious coloring. The first psychiatric hospitals were built in the medieval Islamic world from the 8^th^ century. In the early 8^th^ century, the first hospital was built in Baghdad (705 AD) followed by hospitals built at Fes and Cairo.[7] The first major modern mental hospital, the Bethlehem Hospital, was started/opened in 1247 in London. By the late 18^th^ century, the condition of mentally ill patients in these institutions was one of neglect, restraint and abuse with poor clothing, unhygienic conditions, poor nutrition, restricted movements due to chaining of hands, feet and lack of stimulation, largely contributed to by scarcity of funds, lack of interest among the ruling aristocracy and over-crowding of mental hospitals.[8]

In the late eighteenth and early nineteenth century, Pinel revolutionized care of the mentally ill by propagating a humane approach to care. Around the same time the York retreat was established by William Tuke to provide a kind and tolerant approach towards the mentally ill. Dorothea Dix proposed setting up of State run hospitals for treatment of the mentally ill based upon Pinel’s moral approach.[6] Mid 1950s saw emergence of two major forces which influenced the evolution of modern psychiatry as specific drugs like chlorpromazine were discovered for treatment of mental illnesses; the second being the antipsychiatry movement led by the likes of Goffman, Szaz and others, which along with the economic recession were motivating factors for deinstitutionalization of mentally ill persons and the evolution of the concepts of community psychiatry.[9]

## PSYCHIATRY IN ANCIENT VEDIC INDIA

The descriptions of various mental illnesses in ancient Indian texts are probably the oldest such accounts. Two well-known Ayurvedic manuscripts, the Charaka Samhita by Charaka, and the Sushruta Samhita by Sushruta, have established the roots of modern Indian medicine. The ancient Indian scripture, Atharva-Veda, mentions that mental illness may result from divine curses. Descriptions of conditions similar to schizophrenia and bipolar disorder appear in the Vedic texts. A vivid description of schizophrenia is also found in Atharva-Veda. Other traditional medical systems such as Siddha, which recognize various types of mental disorders, flourished in southern India. Great epics such as the Ramayana and the Mahabharata made several references to disordered states of mind and means of coping with them.[10,11] The Bhagavad Gita is a classical example of crisis intervention psychotherapy. Another interesting contribution of the Ayurveda is its knowledge regarding the diet-disease relationship and the association of a disease with a specific physical constitution. Diagnosis was entertained by the five senses and supplemented by interrogation. According to the ancient system, diagnosis was based on cause (nidana), premonitory indications (purva- rupa), symptoms (rupa), therapeutic tests (upashaya) and natural history of the development of the disease (samprapti). According to Sushruta, the physician (chikitshak), the drug (dravya), the attendants or the nursing personnel (upasthata), and the patient (rogi) are the four pillars on which rests the success of the therapy. The highest patronage to the science of Ayurveda was given by the Buddhist kings (400-200 BC).[4]

Close to the roots of Hindu mythology, Najabuddin Unhammad (1222 AD), an Indian physician propagated the Unani system of medicine as he described seven types of mental disorders; Sauda-a-Tabee (Schizophrenia); Muree-Sauda (depression); Ishk (delusion of love); Nisyan (Organic mental disorder); Haziyan (paranoid state) and Malikholia-a-maraki (delirium). Psychotherapy was known as Ilaj-I-Nafsani in Unani Medicine. The great saga ‘Agastya’ formulated a treatise on mental diseases called as ‘Agastiyar kirigai Nool’, in which 18 psychiatric disorders with appropriate treatment methods were described.[12] Charak Samhita had described various attributes for a hospital including its location, details of equipments, food and cleanliness and model code of conduct for physicians, nursing staff and ward attendants.[13]

The *tridoshic philosophy* is still widely accepted among modern Indian patients. The history of psychiatry in India has witnessed major changes in the past. The first revolution occurred when it was believed that sin and witchcraft are responsible for mental illness and the mentally ill were chained in jails and asylums. Then with the advent of psychoanalysis, etiology of psychiatric disorders was explained. Third was the development of community psychiatry.[14]

## PSYCHIATRY IN PRE-COLONIAL INDIA

During the reigns of King Asoka, many hospitals were established for patients with mental illness. According to the scribes of Asoka Samhita, hospitals were built with separate enclosures for various practices including keeping the patients and dispensing treatments prevailing during those times.[13] A temple of Lord Venkateswara at Tirumukkudal, Chingleput, Tamil Nadu, contains inscriptions on the walls belonging to the Chola period. There are some ancient evidences of propagation of alienation of mentally ill patients in Shahdaula’s Chauhas in Gujarat and Punjab. Though there is not much evidence for development of psychiatry in the Moghul period, there are references to some asylums in the period of Mohammad Khilji (1436- 1469). There is also some evidence of the presence of a mental hospital at Dhar near Mandu, Madhya Pradesh, whose physician was Maulana Fazulur Hakim.[15] There are some historical evidences from the pre-colonial literature that modern medicine and modern hospitals were first brought to India by Portuguese during the seventeenth century in Goa, though documentary evidences are not in good shape to substantiate the claims.[16]

The political instability prevailing in the 1700s saw development of lunatic asylums in Calcutta, Madras and Bombay. It is interesting to observe that these three cities grew up in the beginning largely with British enterprise which conceptualized the segregation of mentally ill patients in mental asylums and their supervision by trained people more in sync with the western conceptualization. The need to establish hospitals became more acute first to treat and manage Englishmen and Indian ‘sepoyees’ employed by the British East India Company.[4] Waren Hastings, the first Governor General, during his regime in 1784 introduced the ‘Pitts India Bill’ according to which the activities of the Government of the East India Company came under the direction of a “Board of Control” and systematic reforms and welfare actions were taken during Lord Cornwallis (1786-93) rule.[17] It was during his rule that there is a reference of the first mental hospital in this part of India at Calcutta recorded in the proceedings of Calcutta Medical Board on April 3, 1787, which became the reference point of inception of colonial influence on development of psychiatric care in India.[15]

## PSYCHIATRY IN COLONIAL INDIA

Ernst (1987) described the growth of mental asylums in British India as a ‘less conspicuous form of social control’.[18] Mental hospitals (or asylums as they were called) in India were greatly influenced by British psychiatry and catered mostly to European soldiers posted in India at that time. Their function was more custodial and less curative.[19]

Development of lunatic asylums was apparent in the early colonial period from 1745 to 1857 till the first revolution for Indian Independence was started. The earliest mental hospital in India was established at Bombay in 1745, which was made to accommodate around 30 mentally ill patients. Surgeon Kenderline started one of the first asylums in India in Calcutta in 1787. Later, a private lunatic asylum was constructed, recognized by the Medical Board under the charge of Surgeon William Dick and rented out to the East India Company.[12] The first government run lunatic asylum was opened on 17 April 1795 at Monghyr in Bihar, especially for insane soldiers.[14] The first mental hospital in South India started at Kilpauk, Madras in 1794 by Surgeon Vallentine Conolly. During this period, excited patients were treated with opium, given hot baths and sometimes, leeches were applied to suck their blood. Music was also used a mode of therapy to calm down patients in some hospitals.[19] The mentally ill from the general population were taken care of by the local communities and by traditional Indian medicine doctors, qualified in Ayurveda and Unani medicine.[16]

The mid-colonial period from 1858-1918 witnessed a steady growth in the development of mental asylums. This period was significant for the enactment of the first Lunacy Act (also called Act No. 36) in the year 1858.[5] The Act was later modified by a committee appointed in Bengal in 1888. During this period, new asylums were also built at Patna, Dacca, Calcutta, Berhampur, Waltair, Trichinapally, Colaba, Poona, Dharwar, Ahmedabad, Ratnagiri, Hyderabad (Sind), Jabalpur, Banaras, Agra, Bareilly, Tezpur and Lahore.[18] Techniques of ‘moral management’ systems which were developed and implemented in this period in the west were also adopted in India. Drug treatments for psychiatric conditions were also introduced into India in this period, e.g., chloral hydrate. These were largely aimed at controlling patient behaviour and also of allowing the patient some respite from his/her condition through sleep.[20] The onset of World War I in 1914 signalled the beginning of a new and distinct period in which strands of continuity were pulled up, in which significant changes took off in the Indian psychiatric system.[14]

Under the Indian Lunacy Act 1912, a European Lunatic Asylum was established in Bhowanipore for European patients, which later closed down after the establishment of the European Hospital at Ranchi in 1918. It was the far-sightedness, hard work and the persistence of the then superintendent of the European Hospital (now known as the Central Institute of Psychiatry), Col Owen A R Berkeley-Hill, that made the institution at Ranchi a unique centre in India at that time which attracted many European patients for treatment. Berkeley-Hill was deeply concerned about the improvement of mental hospitals in those days.[21,22]

The years after 1914 were characterized by gradual expansion rather than building projects and the most significant of these of the period were hangovers from the pre-1914 period. Mental Asylum at Ranchi first opened in 1918 as a hospital for European patients. The sustained efforts of Berkeley-Hill not only helped to raise the standard of treatment and care, but also persuaded the government to change the term ‘asylum’ to ‘hospital’ in 1920.[22] The Parsees during that period were keen to spend large amounts of money to guarantee care in modern psychiatric institutions for those who were considered insane in their own community, often guided by financial rather than therapeutic reasoning.[23] The origins of psychiatric rehabilitation in India can be traced to innovative service programs, which were initiated at the Central Institute of Psychiatry (CIP) in 1922 when Occupational Therapy Unit started at this place. Hydrotherapy started in 1923 and during the same time the hospital started to raise interest of public in mental hygiene and prophylaxis, taking initiatives in preventive aspects of psychiatry.[24] Techniques similar to token- economy were first started in 1920 and called by the name“Habit Formation Chart”.[25] Girindra Shekhar Bose first founded the Indian Psychoanalytical Association in 1922 in Calcutta and Berkeley-Hill started the Indian Association for Mental Hygiene at Ranchi.[22] He was one of the earliest practitioners of psychoanalysis in India who used this technique to help British patients to adjust to their lives after the ravages of World War I.[26] CIP was one of the first centers outside Europe to start Cardiazol-induced seizure treatment in 1938, Electroconvulsive Therapy (ECT) in 1943 and Psychosurgery in 1947. Rauwolfia extracts in the form of Santina, Serpasil and Meralfen were also used for treating psychotic conditions in late 1940s.[22,27]

In the year 1922, CIP got affiliation from the University of London to start Diploma in Psychological Medicine.[22] Grant Medical College, Bombay (now Mumbai) had a Professor of Psychiatry, significantly an Indian, by the year 1936. A memo noted in the archives shows that the number of visits he was to make to the NM Mental Hospital, Thane was to be ‘two per week during the term, when he also gave instructions to the students of the Grant Medical College, Bombay.[5] A library on mental health started in 1918 at CIP with 300 books and journals which dated back to 1910.[22] Child guidance clinic was first established in 1937 at Sir Dorabji Tata Graduate School of Social Work in Bombay.[16] The establishment of Mental Health organization under the Directorate of Health Services was first recommended in 1946 by the health survey and development committee of the Indian Government.[28] The first psychiatric outpatient service, precursor to the present-day general hospital psychiatric units (GHPU), was set up at the R.G. Kar Medical College, Calcutta in 1933 by Ghirinder Shekhar Bose.[8] This was followed by a surge of such units with Masani opening one at JJ Hospital, Bombay in 1938 and Dhunjibhoy opening one day weekly clinic at Prince of Wales Medical College (now Patna Medical College) in 1939.[29]

In 1946, a health survey and development committee, popularly known as the “Bhore Committee,” surveyed mental hospitals. The Health Survey and Development Committee report submitted by Col. Moore Taylor in 1946 reported numerical and professional inadequacy and suggested a focus on training of personnel and students in psychiatry, promotion of occupational and diversionary therapies, and separate child psychiatry units. The committee suggested improvisation and modernization of most hospitals, attachment to medical colleges, and establishment of proper mental health.[28] The World War II saw a separation of military psychiatry from psychiatry in general in India in which the history of modern psychiatry in India seemed to have returned to its origins.

## PSYCHIATRY IN INDEPENDENT INDIA: THE FORMATIVE YEARS

A new phase of development of mental hospitals started after India’s independence in 1947. The government of India focused upon the creation of GHPUs rather than building more mental hospitals. Emphasis was placed upon improving conditions in existing hospitals, while at the same time encouraging outpatient care through these units. A few new mental hospitals, notably at Delhi, Jaipur, Kottayam and Bengal, were added. Mid-1950 witnessed rapid development in the spread to GHPUs in India. In 1957, Dutta Ray started a psychiatric out-patient service at Irwin Hospital (now G.B. Pant Hospital), in New Delhi. In 1958, N.N. Wig started the first GHPU at Medical College, Lucknow, with both in-patient and out-patient psychiatric services and a teaching program as part of the Department of Medicine. Neki started a similar unit at Medical College, Amritsar a few months later. In the next 25 years most of the teaching hospitals and major general hospitals in the private or government sector had GHPUs which were managed by emerging mental health professionals joining services after completing their post graduation in psychiatry.[30]

By the 1960s, traditional institutions like CIP (Ranchi) and Madras Mental Hospital/Asylum offered a range of specialized services, including child and adolescent clinics. Geriatric, epileptic and neuropsychiatric services were added to complete the range of comprehensive OPDs. Another important innovation in the 1960s was the concept of a day hospital. Slowly, alternative accommodations were explored for patients who had recovered, but could not return to their families.[29] CIP started the Department of Clinical Psychology in 1949 which happens to have the first clinical psychology laboratory in the country. CIP also took initiatives in community mental health services as one of the earliest rural mental health clinic was started at Mandar near Ranchi in 1967.

An industrial psychiatric unit was started at Heavy Engineering Corporation (HEC) at Hatia, Ranchi in 1973.[22] Opening of psychiatry units in general hospitals gave psychiatrists an opportunity to demonstrate their knowledge and skills in the management of neurotic and psychosomatic disorders.[30]

On the recommendation of the Bhore committee, All India Institute Mental Health was set up in 1954, which became the National Institute of Mental Health and Neurosciences (NIMHANS) in 1974 at Bangalore. The first training program for Primary Health Care was started in 1978-79.[12] During 1978-1984 Indian Council of Medical Research funded and conducted a multicentre collaborative project on ‘severe mental morbidity’ in Bangalore, Baroda, Calcutta and Patiala. Various training programmes for psychiatrists, Clinical Psychologists, Psychiatric Social Workers, Psychiatric nurses and Primary Care doctors were conducted at Sakalwara unit during 1981-82.[30] Combating stigma and widening the social network of patients were regarded as core elements of a successful rehabilitation programme. During the last 50 years mental health activities have moved from care of the mentally ill to include prevention and promotion of mental health.[31] Keeping with the reforms in community psychiatry, the first psychiatric mental health camp in India was organized in 1972, at Bagalkot, a taluka of Mysore.[12]

Mention must be made of attempts by Wig to use yoga as a therapeutic tool. This period also witnessed efforts to define the core elements of an Indian approach to psychotherapy in the form of a guru-chela relationship.[32] The efforts continued in the 1960s at NIMHANS as there was widespread international acceptance of such approaches, which are known under the rubric of ‘family interventions’.[30]

## PSYCHIATRY IN INDEPENDENT INDIA: ERA OF CONSOLIDATION

As the Government of India embarked on an ambitious national health policy that envisioned “health for all by the year 2000,” early drafts of the National Mental Health Program were formulated, subsequently adopted by the Central Council of Health and Family Welfare, in 1982. Since its inception, there has been development of a model District Mental Health Program, and development of training materials and programs for practitioners and academicians.[33]

The first draft of Mental Health Act that subsequently became the Mental Health Act of India (1987) was written at Ranchi in 1949 by R.B. Davis, then Medical Superintendent of CIP, S.A. Hasib, from Indian Mental Hospital, Ranchi and J Roy, from Mental Hospital, Nagpur.[22] Initial attempts by the Indian Psychiatric Society to bring about change were unsuccessful. In 1959-60, reforms were considered but no consensus was reached. In the 1980s, there was a resurgence of activity resulting in the passage of the Mental Health Act in 1987.[22,34]

### The Erwadi tragedy

In 2001 a horrific incidence took place at Erwadi in which 26 persons with mental illness died in a tragic fire accident. The response of the general population, the administrators, the politicians, the press and the professionals was one of shock and outrage. The press seized the moment and wrote about similar situations, in Hyderabad, Ranchi, Ahmedabad, and Patiala. The National Human Rights Commission called for a Report. The Supreme Court initiated action on the matter. As a result, many changes not only in Erwadi but also in the different parts of the country started taking shape, which proved to be a yardstick which revamped mental health services in the country.[35]

Research in psychiatry started rolling with commencement of publication of first journal dedicated to mental health, The “Indian Journal of Neurology and Psychiatry” in 1949. The Indian Journal of Psychiatry started in 1958 and has now completed 50 golden years of continuous enrichment in the field of psychiatry in India.[36] The journal got indexed in National Library of Medicine, the Catalogue of Index Medicus as the present review has been written in 2009. Psychoanalytically oriented literature and theoretical texts dominated the research literature from 1947 to 1960. During the second phase of psychiatric research (1960- 1972), a distinctive trend emerged as research publications moved from individual psychopathology to the interface between the individual and society and group behaviour.

Among the major epidemiological studies of the early days included those of Surya, Sethi, Ganguli and Gopinath, which helped to establish the magnitude of mental health problems in the community. Mental health researchers in this decade were also active in the field of psychological testing.[37] Clinical studies form a substantial bulk of research in last 25 years. The year 1980 saw a fresh surge in mental health research programmes as many projects were started in various parts of the country in collaboration with Indian Council of Medical Research and World Health Organization (WHO). The researchers in last two decades have matured, and studies on diverse subjects including mental health in children, have been published. Biological psychiatry has been a woefully neglected area in Indian research though in recent years some original work has been published, but it is nowhere near the contemporary work from West.[38]

The Mudaliar Committee also noted the serious shortage of trained mental health manpower and recommended the development of the European Mental Hospital at Ranchi (now CIP) into a full-fledged training institute. A formal training program for clinical psychologists (Diploma in Medical Psychology) also commenced at NIMHANS in the year 1955 and was later converted into an M. Phil in Medical and Social Psychology in 1978. In keeping with the recommendations of the Mudaliar Committee, the Central Institute of Psychiatry started training for clinical psychologists in 1962.[22]

Girinder Shekhar Bose founded the Indian Psychoanalytical Association in 1922 in Calcutta. Berkeley-Hill, in 1929, founded the Indian Association for Mental Hygiene. D. Satyanand was another analyst who received his personal analysis by Berkeley-Hill. In 1935, the Indian division of the Royal Medico-Psychological Association was formed due to the efforts of Banarasi Das. In 1946, Nagendra Nath De consulted R. B. Davis of the European Mental Hospital, Ranchi and T. A. Munro, an advisor in Psychiatry to the Indian Army and decided to revive the association.[36] The decision to form the Indian Psychiatric Society, the national organization of psychiatrists in India was taken in the meeting convened by R.B. Davis in Delhi on 7^th^ January 1947 during the annual congress of Indian Science Congress at Delhi University.[36]

## CONCLUSION

The amalgamation of mental health, primary health care has led to a major shift from the concept of custodial care to one that emphasizes on care and treatment, although a huge gap between the rhetoric of this new policy and its implementation still remains. Mental hospitals, with all their inherent flaws and drawbacks, are powerful institutions for the proper care of a subset of mentally ill persons, especially those with severe forms of illness and poor familial/social supports.[33]

The last two decades have seen an explosion in the knowledge base of the neurosciences, epidemiology and therapeutics. There has also been a parallel growth in interdisciplinary linkages, which support integrated socially and culturally appropriate approaches to mental health interventions. It is sometimes difficult for contemporary practitioners to fully comprehend the wide ranging challenges that confronted mental health professionals in the period following India’s independence. However, it is important to remember that the foundations for the current knowledge base were laid during those early years.
